# Free Linoleic Acid and Oleic Acid Reduce Fat Digestion and Absorption In Vivo as Potent Pancreatic Lipase Inhibitors Derived from Sesame Meal

**DOI:** 10.3390/molecules27154910

**Published:** 2022-08-01

**Authors:** Xuan Li, Sayo Morita, Hiroaki Yamada, Keita Koga, Wakana Ota, Toma Furuta, Atsushi Yamatsu, Mujo Kim

**Affiliations:** 1 Pharma Foods International Co., Ltd., Kyoto 615-8245, Japan; s-morita@pharmafoods.co.jp (S.M.); h-yamada@pharmafoods.co.jp (H.Y.); k-koga@pharmafoods.co.jp (K.K.); mujokim@pharmafoods.co.jp (M.K.); 2 Mitsui Sugar Co., Ltd., Tokyo 103-8423, Japan; wakana.oota@mitsui-sugar.co.jp (W.O.); toma.furuta@mitsui-sugar.co.jp (T.F.)

**Keywords:** pancreatic lipase inhibitor, functional molecule, free fatty acids, oleic acid, linoleic acid, sesame, obesity, fat digestion and absorption, dietary supplement

## Abstract

Pancreatic lipase catalyzes the cleavage of triacylglycerols at the oil–water interface, and is known as the dominant determiner of dietary fat digestion. Reducing dietary fat digestion and absorption by modulating the activity of pancreatic lipase has become a favorable strategy to tackle obesity. Orlistat is, at present, the only pancreatic lipase inhibitor approved for the treatment of obesity; however, an array of gastrointestinal adverse effects associated with orlistat limits its tolerability. As a safe alternative to orlistat, a number of natural product-derived compounds with varying degrees of pancreatic lipase inhibitory activity have been reported. We herein reported that bioactivity-guided fractionation of sesame meal led to the identification of free linoleic acid and oleic acid as potent inhibitors of porcine pancreatic lipase in vitro with an IC50 of 23.1 µg/mL (82.4 µM) and 11.7 µg/mL (41.4 µM), respectively. In rats, a single oral dose of the mixture of these fatty acids significantly suppressed the elevation of blood triacylglycerol level following fat intake. These results substantiate the role of free linoleic acid and oleic acid as a novel class of natural product-derived functional molecules that act as pancreatic lipase inhibitors, and their potential for healthy, routine-based weight management.

## 1. Introduction

The prevalence of obesity continues to rise at an alarming rate worldwide, with nearly 40% of adults and almost one in five children and adolescents described as overweight or obese, according to the latest World Health Organization (WHO) Global Health observatory data collated in 2016 [1]. Obesity increases the risk of a range of metabolic and cardiovascular diseases, some cancers, and severe illness and death from COVID-19 [2]. Obesity affects nearly all age groups and constitutes a massive economic burden on healthcare systems. Dietary energy intake is a major contributor to and one of the modifiable risk factors for obesity. In most populations, fat intake accounts for 30% or more of the total energy intake, which exceeds the recommended intake of fats for the prevention of noncommunicable diseases [3]. It is evident that energy intake and the risk of obesity increase as dietary fat content increases [4,5,6].

The digestion and absorption of dietary fat, predominantly triacylglycerols (TAGs), consist of sequential physicochemical and enzymatic events through the gastrointestinal tract. TAGs are digested primarily by pancreatic lipase (triacylglycerol acyl hydrolase EC3.1.1.3) in the upper segment of the jejunum. The enzyme is secreted by the acinar glands of the pancreas into the pancreatic duct and then into the intestine in response to the ingestion of a fatty meal. At the oil–water interface, pancreatic lipase acts on the sn-1 and sn-3 positions of TAG molecules, resulting in the release of 2-monoacylglycerol and free fatty acids, which are taken up from the lumen into the polarized enterocytes that line the small intestine and used for the biosynthesis of neutral fat. Further hydrolysis of 2-monoacylglycerol by pancreatic lipase results in the formation of glycerol and free fatty acids [7]. Pancreatic lipase is known as the single most important determinant of dietary fat absorption, as in its absence only 30% of ingested fats are absorbed [8]. Hence, modulating human pancreatic lipase may represent an effective way to combat obesity.

Orlistat, a potent inhibitor of gastric and pancreatic lipases, is now the only medication approved for clinical use to aid weight loss, and the only anti-obesity medicine that does not act on the central nervous system or enter the bloodstream [9]. However, orlistat has been associated with fat malabsorption [10] and multiple gastrointestinal adverse effects, which compromise patients’ compliance, and drug interactions that affect its bioavailability and effectiveness [11]. Moreover, since orlistat is indicated for patients with a body mass index (BMI) >30 or those with a BMI >27 and concomitant obesity-related risk factors or diseases [10], it means that orlistat can hardly be used by healthy individuals on a regular basis for weight management purposes. Alternatively, bioactive compounds of natural origins that target pancreatic lipase have received substantial attention owing to their structural diversity, low toxicity, and wide range of sources.

During the process of fractioning sesame meal, a byproduct of sesame (*Sesamum indicum* L.) oil extraction, we fortuitously found that the ethanolic extract of sesame meal effectively inhibited the activity of porcine pancreatic lipase in in vitro assays. Therefore, we proceeded to identify the bioactive constituent(s) of sesame meal against pancreatic lipase, and to explore its/their effectiveness in reducing dietary fat absorption in vivo.

## 2. Results

### 2.1. Identification of the Pancreatic Lipase Inhibitory Constituents of Sesame Meal

**Figure 1:**
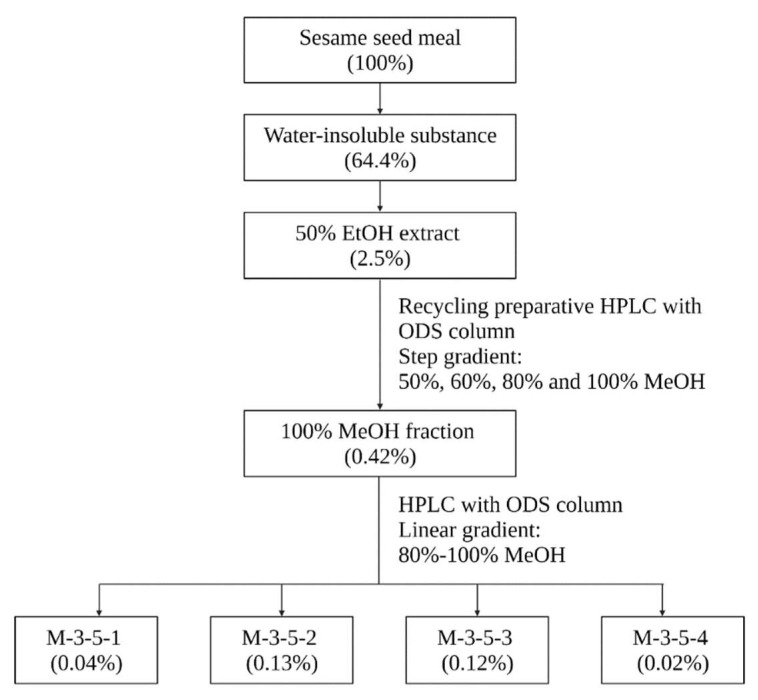
Flow chart illustrating the extraction, fractionation, and purification of pancreatic lipase-inhibitory compounds from sesame meal by sequential solvent extraction and HPLC. The percentages in parentheses denote the yields. EtOH: ethanol; MeOH: methanol.

The fractionation process of sesame meal and the yields of each fraction are illustrated in Figure 1.

**Figure 2:**
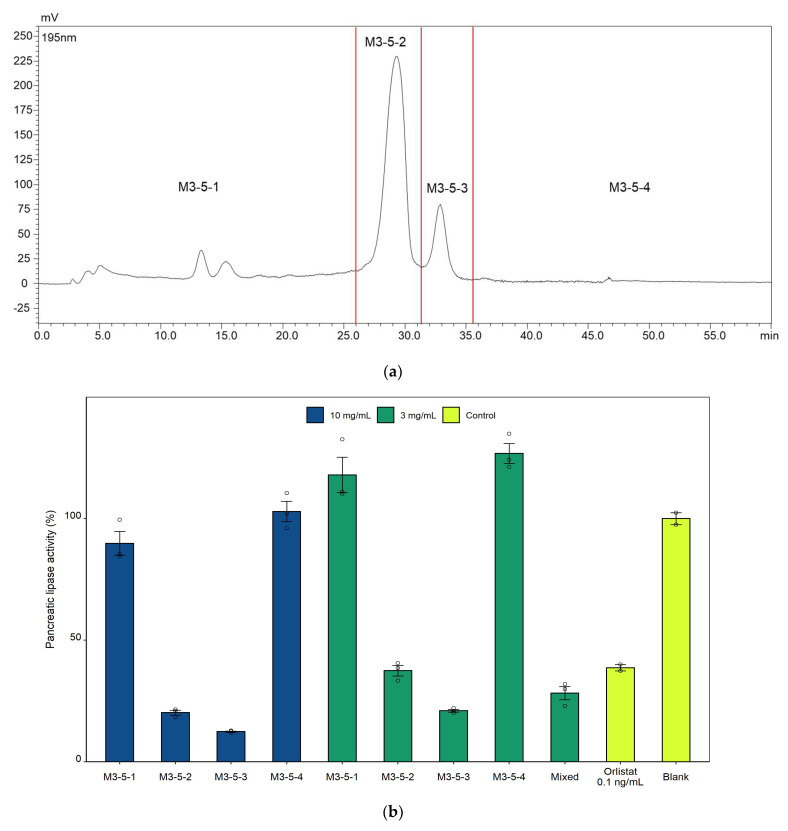
In vitro pancreatic lipase activity of the fractions derived from sesame meal. (**a**) Fractionation of the 100% methanolic fraction with HPLC on a polymeric ODS column afforded four fractions (M3-5-1, 2, 3, and 4). (**b**) Two (M3-5-2 and M3-5-3) of the four fractions in (**a**) displayed high potency against pancreatic lipase. The concentrations of fraction M3-5-1, 2, 3, and 4 were equivalent to 10 mg/mL (blue bar) and 3 mg/mL (green bar) of the 50% ethanol extract.

Initially, lipase inhibition assay with the crude extracts of sesame meal revealed that the 40 °C 50% ethanolic extract reduced the pancreatic lipase activity by 92.7% at 3 mg/mL. Subsequent fractionation of the ethanolic extract afforded four fractions, among which the 100% methanol fraction exhibited the highest inhibitory activity, accounting for 91.6% of the total inhibition exerted by the ethanolic extract. The 100% methanol fraction was clarified and further fractionated, affording four fractions (Figure 2a), among which two fractions (M3-5-2 and M3-5-3), at concentrations equivalent to 10 mg/mL and 3 mg/mL of the 50% ethanol extract, exhibited significant inhibitory effects on pancreatic lipase activity (Figure 2b).

**Figure 3:**
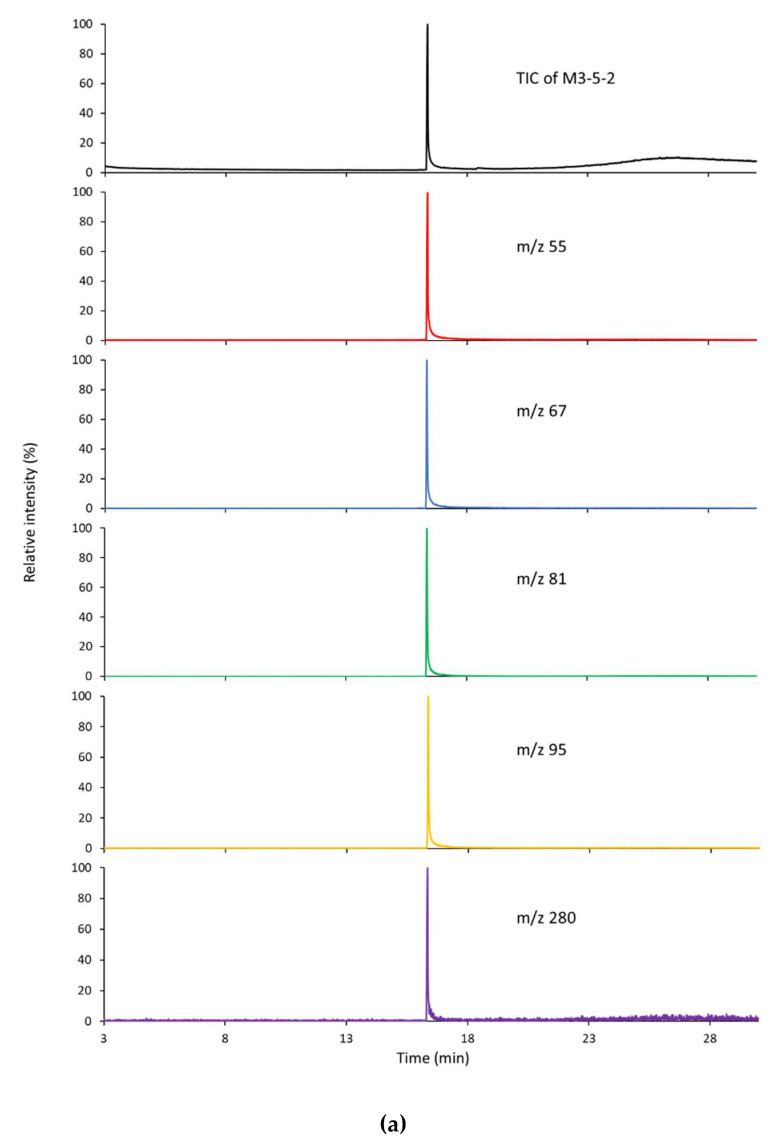

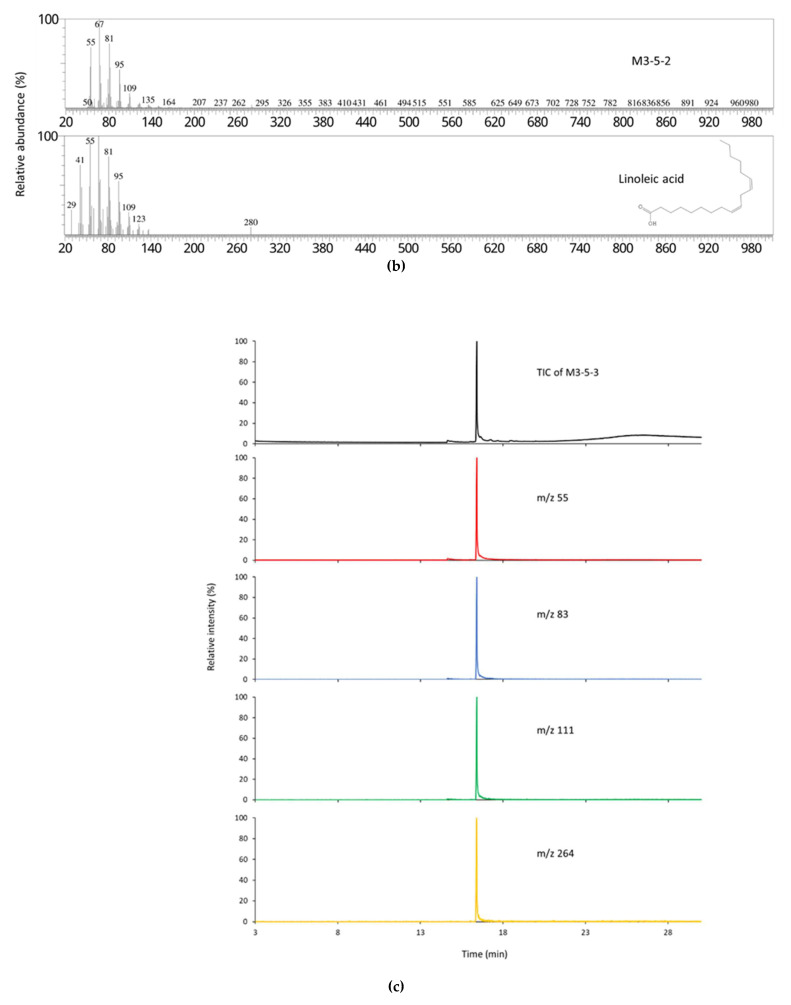

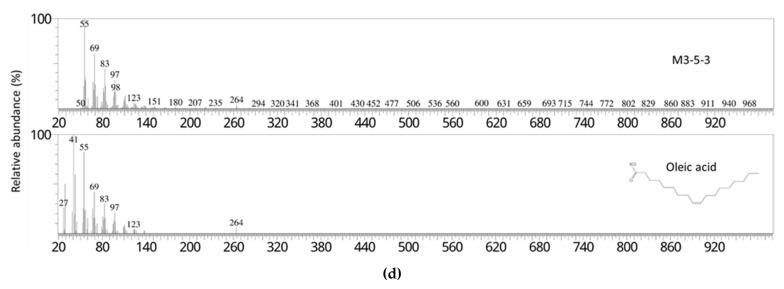
GC-MS analysis of the bioactive fractions against pancreatic lipase in vitro. (**a**) The TIC and EIC of fraction M3-5-2. (**b**) Mass spectra of fraction M3-5-2 (upper panel) and linoleic acid (lower panel). (**c**) The TIC and EIC of fraction M3-5-3. (**d**) Mass spectra of fraction M3-5-3 (upper panel) and oleic acid (lower panel).

**Figure 4:**
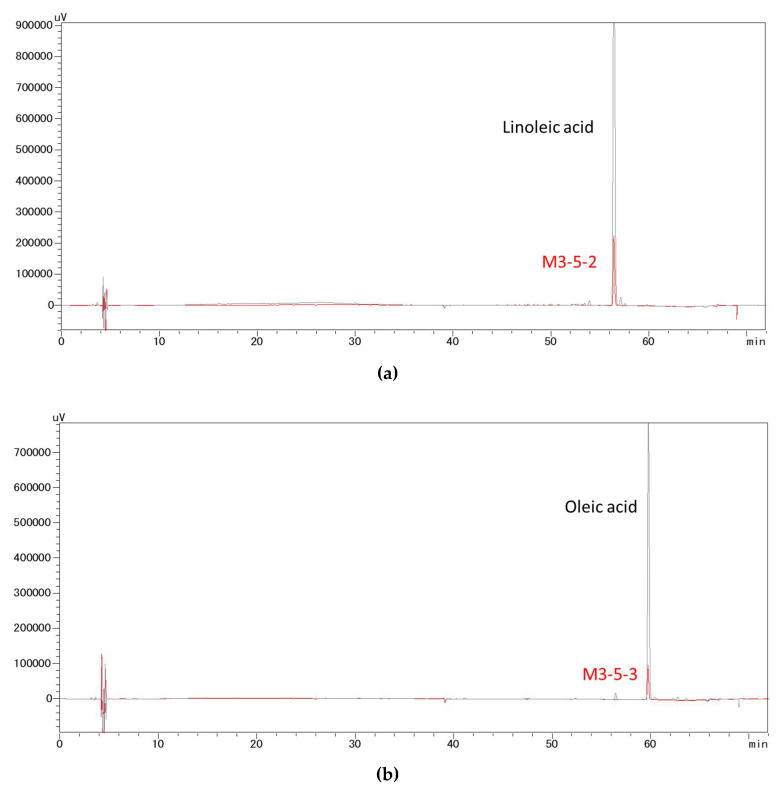
HPLC chromatogram of the bioactive fractions against pancreatic lipase in vitro. (**a**) Fraction M3-5-2 and the linoleic acid standard. (**b**) Fraction M3-5-3 and the oleic acid standard.

The GC-MS chromatograms and mass spectra of fraction M3-5-2 and M3-5-3 are shown in Figure 3. The total ion chromatogram (TIC) and extracted ion chromatogram (EIC) suggest that both fractions are predominantly composed of one component (Figure 3a,c). By matching the mass spectrum of fraction M3-5-2 (Figure 3b) with the reference spectra in the National Institute of Standards and Technology (NIST) library, the peak of fraction M3-5-2 was identified to be linoleic acid. For fraction M3-5-3, the mass spectrum of the prominent peak matched that of oleic acid (Figure 3d). These compounds were further confirmed by matching their HPLC retention times with those of authentic compounds. Specifically, fraction M3-5-2 had a retention time of 56 min, which was in accordance with that of linoleic acid standard (Figure 4a), whereas M3-5-3 had a retention time of 60 min, which agreed with that of oleic acid (Figure 4b).

### 2.2. Linoleic Acid and Oleic Acid Inhibited Porcine Pancreatic Lipase Activity In Vitro

**Figure 5:**
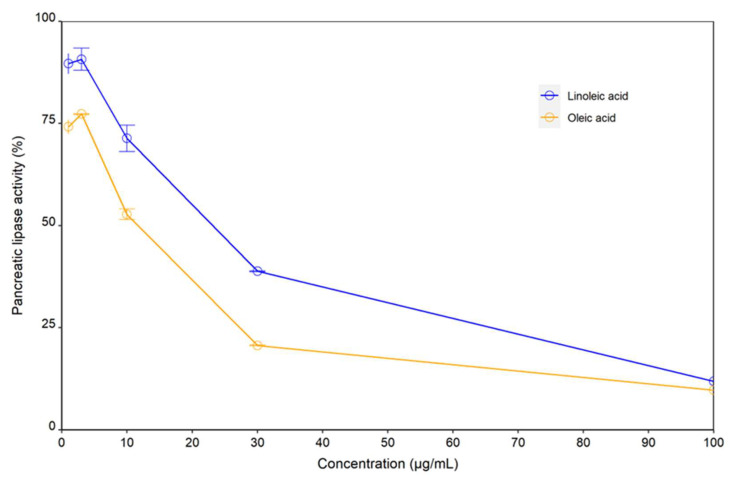
In vitro inhibitory activity of linoleic acid and oleic acid against pancreatic lipase at varying concentrations. Linoleic acid and oleic acid exhibited an IC50 of 23.1 µg/mL (82.4 µM) and 11.7 µg/mL (41.4 µM), respectively.

The hydrolytic activity of porcine pancreatic lipase was determined in the presence of increasing concentration of linoleic acid and oleic acid, respectively (Figure 5). Both linoleic acid and oleic acid were revealed to inhibit pancreatic lipase activity in vitro in a concentration-dependent manner. Oleic acid exhibited higher degrees of inhibition over the entire concentration range with an IC50 of 11.7 µg/mL (0.04 µM/mL), comparing to linoleic acid, which showed an IC50 of 23.1 µg/mL (0.08 µM/mL).

### 2.3. Linoleic Acid and Oleic Acid Combined to Lower Postprandial TAGs in High-Fat Diet (HFD)-Challenged Rats

**Figure 6:**
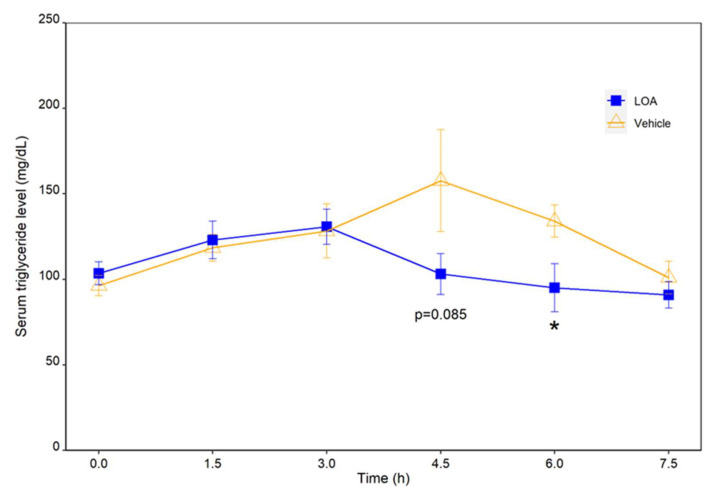
Post-prandial TAG responses of rats administrated with a mixture of linoleic acid and oleic acid (LOA) at a dose of 0.46 mg/kg and 0.72 mg/kg body weight or the vehicle and challenged with an HFD. * *p* < 0.05.

The trajectories of blood TAG levels in the rats administered with the vehicle or a mixture of linoleic acid and oleic acid (LOA) following HFD challenge are presented in Figure 6. The blood TAG levels of both diet groups increased slightly within the first 3 h postprandial. After this point, the TAG level of the LOA group started to decline rapidly, and was restored to the preprandial level at 4.5 h after HFD administration, whereas that of the vehicle group continued to rise until 4.5 h before turning downward and did not return to the preprandial level until 7.5 h. As a result, the mean TAG level of the LOA group at 4.5 h was considerably lower than that of the vehicle group (*p*-value = 0.085), though statistical significance was not achieved. Nonetheless, the LOA-treated rats exhibited a significantly reduced blood level of TAG at 6 h postprandial compared to their counterparts that received the vehicle only (*p*-value = 0.042).

## 3. Discussion

Reducing dietary fat digestion and absorption by modulating the activity of pancreatic lipase has become a favorable strategy to tackle obesity, not only because pancreatic lipase is the single most important determinant of fat absorption, but targeting pancreatic lipase is also considered safe, as its inhibitors neither act on the central nervous system nor enter the bloodstream. At the present time, the only approved anti-obesity drug that acts by inhibiting pancreatic lipase is orlistat, which, however, is known to have multiple gastrointestinal side effects. As a safe alternative to orlistat, a number of natural product-derived compounds with varying degrees of inhibitory activity against pancreatic lipase have been identified. These compounds are derived from a wide range of plant and microbial extracts and belong to various chemical classes, including, but not limited to, alkaloids, carotenoids, polyphenols, glycosides, polysaccharides, terpenes, and saponins [9,12,13]. In this regard, for instance, strictinin-rich white tea and green tea extracts were found to be effective in inhibiting pancreatic lipase in vitro (IC50 = 22 μg/mL and 35 μg/mL, respectively) [14]; stephalagine (1,2-methylenedioxy-3-methoxyaporphine), an aporphine alkaloid isolated from *Annona crassiflora* fruit peel, demonstrated high potency against pancreatic lipase, with an IC50 of 8.35 µg/mL (27.0 µM) [15]; for the methanolic extracts of *Eucalyptus globulus* and *Mentha viridis*, the pancreatic lipase inhibitory activity (IC50 = 690 µg/mL and 1290 µg/mL, respectively) seemed to hinge on the contents of phenols and total flavonoids [16]; the fractionation of *Murraya koenigii* (L.) Spreng leaves led to the identification of four carbazole alkaloids that exhibited pancreatic lipase inhibitory activities, with IC50 values ranging from 17.9 µM to < 500 µM [17].

In this study, bioactivity-guided fractionation of the 40 °C 50% ethanol extract of sesame meal afforded two fractions that exerted the most potent in vitro inhibitory effects on pancreatic lipase. These fractions were suggested to comprise predominantly linoleic acid and oleic acid, respectively, by matching their fragmentation pattern and retention time with those of the pure analytical standards. As confirmed in the subsequent lipase inhibition assays, both linoleic acid and oleic acid demonstrated strong pancreatic lipase activities in vitro, which were comparable or superior to a number of pancreatic lipase inhibitors derived from natural sources, including the aforementioned ones. However, direct and rigorous comparison between these extracts or compounds is difficult due to the differences in the source of the enzyme and assay conditions. In addition to lipase inhibition assay, to further clarify the mechanism of action of free linoleic acid and oleic acid, molecular docking simulations and inhibition kinetic analysis will be needed in future studies.

We then proceeded to verify the combined effectiveness of linoleic acid and oleic acid in vivo on the digestion and absorption of dietary TAGs by monitoring the postprandial TAG responses in rats to an oral administration of these fatty acids along with an HFD. This experiment is based on the well-established notion that effective intestinal absorption of TAGs depends upon their hydrolysis catalyzed by pancreatic lipase. As a result, an oral administration of a mixture of linoleic acid and oleic acid at a dose of 0.46 mg/kg and 0.72 mg/kg body weight significantly suppressed the postprandial TAG increase, and led to a faster restoration of TAG to the preprandial level in HFD-challenged rats, as compared to the vehicle-treated group. These results suggested that the linoleic-oleic acid mixture used in the present study was effective in reducing the absorption of TAGs into circulation following an oral fat load. This coincided with the finding that these fatty acids were effective pancreatic lipase inhibitors in vitro, and thus it is reasonable to speculate that these fatty acids attenuate postprandial triglyceridemia by targeting pancreatic lipase, the single most important determinant of lipid absorption. Moreover, for the individual free fatty acids, as well as for the cocktail of these free fatty acids, the profile of safe and effective doses and the dose–response relationship remain to be established in future research.

Linoleic acid and oleic acid are the two most abundant dietary plant-derived fatty acids, and they collectively constitute over 80% of the total fatty acids of sesame seeds [18]. Oleic acid is also the single most common monounsaturated fatty acid (MUFA) in daily nutrition, whereas linoleic acid is an essential n-6 polyunsaturated fatty acid (PUFA) widely found in plant oil. Recently, oleic acid has attracted much attention owing to its effectiveness in regulating food intake, body mass, and energy metabolism by modulating multiple cellular pathways, as reviewed by Tutunchi et al. [19]. Linoleic acid has been reported to reduce blood cholesterol and to play a vital role in preventing cardiovascular diseases [7]. Previously, fatty acids were suggested to be one of the bioactive components of the pancreatic lipase inhibitory *Arachis hypogaea* nutshell extract [20]. However, in this study, the authors did not specify which fatty acids were involved, in which form (esterified or free) these fatty acids existed and exhibited their inhibitory activity, or the individual effects of each fatty acid. Later, linoleic acid and oleic acid, along with other fatty acids and phytochemical compounds, were suggested to contribute to the pancreatic lipase inhibitory activity of *Elateriospermum tapos* seed [21]. Recently, linoleic acid (along with palmitic acid and linolenic acid) was identified in a free fatty acid-enriched dichloromethane extract of *Mimosa diplotricha* bee pollen, which exhibited potent porcine pancreatic lipase inhibitory activity in vitro (IC50 of 52.6 µg/mL). However, in these studies, the inhibitory activities of individual fatty acids were not evaluated or verified, and the in vivo effectiveness of these fatty acids or the bioactive extracts in reducing fat digestion and absorption remained uninvestigated [22]. In this study, we demonstrated for the first time that linoleic acid and oleic acid were not only potent inhibitors of pancreatic lipase in vitro, but also effective in reducing intestinal absorption of dietary TAGs in vivo, adding a new facet to the health benefits of these fatty acids. Moreover, it is worth noting that fatty acids are predominantly present in their esterified forms in dietary fats, whereas, as reported herein as well as in [22], the fatty acids exerted their inhibitory effects on the digestion of fats in their non-esterified or free forms. In line with this, as intestinal digestion of dietary TAGs releases free fatty acids, it is possible that pancreatic lipase encounters increasing levels of inhibition by its products. Such product inhibition might affect the dynamics of pancreatic lipase-catalyzed fat digestion, which warrants further investigation.

Through this study, we have widened the spectrum of pancreatic lipase inhibitors derived from natural sources to further include members of free mono- and poly- unsaturated fatty acids. In particular, we demonstrated that free linoleic acid and oleic acid were effective inhibitors of pancreatic lipase and modulators of fat digestion and absorption, and justified their potential use as regulators of energy intake in the management of overweight and obesity. Owing to their great abundance in food materials, not only do linoleic acid and oleic acid have minimal adverse effects in contrast to orlistat, but they are also readily incorporable into the diet and hence the effective dose is easily achievable. These advantages warrant further investigation on the effectiveness of free linoleic acid and oleic acid and the utilization of natural products rich in these fatty acids in reducing triglyceride absorption in rodent models and its translation to human research. Additionally, as free fatty acids have low solubility in water, nanotechnology formulations of these molecules may represent an effective strategy to facilitate their incorporation into aqueous matrices and to enhance their in vivo performance against the water-soluble pancreatic lipase.

## 4. Materials and Methods

### 4.1. Chemicals

All the chemicals used for this study were of analytical grade. Linoleic acid (18:2 cis-9,12), oleic acid (18:1 cis-9), anhydrous pyridine, ethanol, methanol, and formic acid were purchased from FUJIFILM Wako Pure Chemical Corporation (Kyoto, Japan). Acetonitrile was purchased from Nacalai Tesque (Kyoto, Japan).

### 4.2. Extraction and Fractionation of Sesame Meal

The sesame meal used in this study was a byproduct of sesame oil pressing, and was provided by Mitsui Sugar Co., Ltd. The sesame meal was firstly extracted four times with 10 volumes (*v/w*) of distilled water by stirring at room temperature for 1 h. The resulting solid residues were collected and lyophilized. The lyophilized powder was then extracted twice with 10 volumes (*v/w*) of 50% ethanol at 40 °C for 3 h. The soluble part was recovered and lyophilized after the solvent was reduced using a rotary evaporator. The 50% ethanol extract was further fractionated by using recycling preparative high-performance liquid chromatography (HPLC) with an octadecyl silica (ODS) column. In this regard, the 50% ethanol extract was redissolved in 10 times (*v/w*) of 50% ethanol at room temperature, aliquoted into 1.5 mL tubes, and centrifuged at 10,000 rpm for 3 min. The supernatant was then clarified with 0.45 μm filter, and 3 mL of the filtrate was applied to the recycling preparative HPLC (LC908, Japan Analytical Industry). The chromatographic separation was performed on a Biotage^®^ Sfär C18D Duo column (100 Å, 30 μm, 30 g, 45 mL). UV detection was carried out at 195 nm. The filtrate was recycled and eluted stepwise at 7.5 mL/min with 50% methanol (7.9 column volume (CV), 1CV = 45 mL), 60% methanol (5CV), 80% methanol (5CV), and 100% methanol (5CV). The 100% methanol fraction, which exerted the highest inhibitory activity against pancreatic lipase, as revealed by the in vitro lipase inhibition assay, was clarified and further separated with a Biotage^®^ Sfär C18D Duo column (100 Å, 30 μm, 12 g) in a Shimadzu HPLC system with a linear methanol gradient from 80% to 100% over 30 min at a constant flow rate of 2.5 mL/min. UV detection was carried out at 195 nm. These fractions were then subject to the lipase inhibition assay.

### 4.3. Lipase Inhibition Assay

Pancreatic lipase solution was prepared by vigorously mixing 30 mg of lipase from porcine pancreas Type II (Sigma) with 30 mL 0.1 M citrate buffer (pH 6.0) in an ice bath for 1 h. The mixture was centrifuged at 4 °C, 10,000 rpm for 45 min, and the supernatant was recovered and diluted to 0.06 mg/mL as the working enzyme solution.

The activity of the extracts and fractions of sesame meal, linoleic acid, and oleic acid against pancreatic lipase was evaluated by using the Lipase Kit S (SB Bioscience, Osaka, Japan) per the manufacturer’s instructions. Specifically, the lyophilized extracts and fractions were dissolved in DMSO and then diluted 10 times with distilled water. 0.1 ng/mL of orlistat (prepared in 10% (*v/v*) DMSO) was used as a positive control. For blank measurements, 10% (*v/v*) DMSO was used. 100 μL of the sample, 237 μL of the color-developing agent (5,5′-dithiobis-(2-nitrobenzoic acid), DTNB), 9 μL of the prepared pancreatic lipase solution, and 4 μL of the esterase inhibitor (phenylmethylsulfonyl fluoride, PMSF) were mixed and incubated at 30 °C for 5 min, followed by the addition of 25 μL of substrate solution (2,3-dimercapto-1-propanol-tributyrate, BLAB) and incubation in darkness for 30 min. The reaction was quenched by adding 700 μL of stop solution, and the absorbance was measured at 412 nm. Background absorbance caused by the reaction components was determined by incubating 100 μL of the sample with 237 μL of the color-developing agent, 9 μL of the prepared lipase solution, and 4 μL of the esterase inhibitor in darkness for 35 min, followed by the addition of 700 μL of stop solution and 25 μL of substrate solution. The absorbance at 412 nm was then measured.

The percentage inhibition of pancreatic lipase activity by each sample was calculated using the following equations:

Pancreatic lipase activity (%) = (Sample OD412 − Background OD412) × 100/(Blank OD412 − Blank of background OD412);

Percentage inhibition of pancreatic lipase activity (%) = 100 − Pancreatic lipase activity (%).

### 4.4. Identification of the Bioactive Constituents against Pancreatic Lipase

The bioactive constituents of sesame meal against pancreatic lipase were identified by using gas chromatography-mass spectrometry (GC-MS) and HPLC, and by fragmentation pattern and retention time matching with the reference compounds.

For GC-MS analysis, 0.1 mg of the sample was dissolved in 20 μL of pyridine, and 1 μL was injected into a Shimadzu GCMS-QP2010 Plus system with a UA-5 (MS/HT) column (Ultra ALLOY) at an inlet split ratio of 1:50. Helium was used as the carrier gas, and the flow rate was 1.0 mL/min. The column temperature was programed to increase from 80 °C at 10 °C/min to 330 °C with a hold time of 5 min. A mass range of 50 *m/z*–1000 *m/z* was scanned.

HPLC analysis was performed on a Shimadzu HPLC system with a YMC-Pack ODS-A column (12 nm, 5 µm, 250 mm × 4.6 mm). A binary gradient elution was employed, i.e., 0.1% (*v/v*) formic acid aqueous solution and acetonitrile with 0.1% (*v/v*) formic acid, with the proportion of 0.1% formic acid increasing from 1% to 100% in 60 min at a flow rate of 0.8 mL/min and under 40 °C. UV detection was carried out at 195 nm.

### 4.5. Animal Experiments

All procedures involving experimental animals were performed in accordance with the “Basic guidelines for the conduct of animal experiments in implementing agencies under the jurisdiction of the ministry of health, labor and welfare”, and followed the protocols approved by the Committee on Animal Research and Ethics of Pharma Foods International Co., Ltd. (19KRD-008).

Six-week-old, male Wistar rats were purchased from Japan SLC, Inc. All rats were singly caged at 23 °C and 55% humidity with ad libitum access to water and a standard rodent diet (MF laboratory chow, Oriental Yeast Co., Tokyo, Japan) for the first 5 days following delivery to allow acclimation to the new environment. The rats were maintained on a 12:12-h light–dark cycle throughout the study. After acclimation, the rats were divided into two diet groups (7 rats per group; groups matched for body weight) and fasted overnight (16 h) prior to the HFD challenge.

The HFD used in the present study was a corn-oil-based lipid emulsion, which was prepared by ultrasonically emulsifying 30 mL of corn oil, 400 mg of cholic acid, 10 g of cholesterol oleate, and 30 mL of water.

Linoleic acid and oleic acid were dissolved in 400 μL of 20% (*v/v*) ethanol (vehicle), and administrated at a dose of 0.46 mg/kg and 0.72 mg/kg body weight, respectively. The LOA or the vehicle was administered to rats via gastrostomy tubes 15 min prior to the administration of the lipid emulsion (0 h). Blood samples were obtained from the tail vein at 0 h, 1.5 h, 3 h, 4.5 h, 6 h, and 7.5 h. Blood samples were centrifuged at 2500 rpm for 10 min, and the sera were stored at −20 °C until analysis. The quantification of TAGs in the serum was performed by using a LabAssayTM Triglyceride Kit (Wako) per the manufacturer’s instructions.

### 4.6. Statistical Analysis

Data are presented as mean ± standard error (S.E.), unless indicated otherwise. A two-tailed, Welch’s t-test was used to test for between-group differences. One-way ANOVA with Tukey’s HSD (honestly significant difference) tests were used for multiple comparisons. Differences at a probability level (*p*-value) of 0.05 were considered statistically significant.

## Data Availability

Not applicable.

## References

[1] World Health Organization (WHO), obesity and overweight. https://www.who.int/news-room/fact-sheets/detail/obesity-and-overweight.

[2] Wise J. (2021). Covid-19: Highest death rates seen in countries with most overweight populations. BMJ.

[3] Joyce P., Meola T.R., Schultz H.B., Prestidge C.A. (2020). Biomaterials that regulate fat digestion for the treatment of obesity. Trends. Food. Sci. Technol..

[4] Donahoo W., Wyatt H.R., Kriehn J., Stuht J., Dong F., Hosokawa P., Grunwald G.K., Johnson S.L., Peters J.C., Hill J.O. (2008). Dietary fat increases energy intake across the range of typical consumption in the United States. Obesity. (Silver Spring).

[5] Hill J.O., Melanson E.L., Wyatt T.H. (2000). Dietary fat intake and regulation of energy balance: Implications for obesity. J. Nutr..

[6] Hu S., Wang L., Yang. D., Li L., Togo J., Wu Y., Liu Q., Li B., Li M., Wang G. (2018). Dietary fat, but not protein or carbohydrate, regulates energy intake and causes adiposity in mice. Cell Metab..

[7] Farvid M.S., Ding M., Pan A., Sun Q., Chiuve S.E., Steffen L.M., Willett W.C., Hu F.B. (2014). Dietary linoleic acid and risk of coronary heart disease: A systematic review and meta-analysis of prospective cohort studies. Circulation.

[8] Birari R.B., Bhutani K.K. (2007). Pancreatic lipase inhibitors from natural sources: Unexplored potential. Drug Discov..

[9] Liu T., Liu X., Chen Q., Shi Y. (2020). Lipase inhibitors for obesity: A review. Biomed. Pharmacother..

[10] Heck A.M., Yanovski J.A., Calis K.A. (2000). Orlistat, a new lipase inhibitor for the management of obesity. Pharmacotherapy.

[11] Filippatos T.D., Derdemezis C.S., Gazi I.F., Nakou E.S., Mikhailidis D.P., Elisaf M.S. (2008). Orlistat-associated adverse effects and drug interactions. Drug Saf..

[12] Buchholz T., Melzig M.F. (2015). Polyphenolic compounds as pancreatic lipase inhibitors. Planta Med..

[13] Lunagariya N.A., Patel N.K., Jagtap S.C., Bhutani K.K. (2014). Inhibitors of pancreatic lipase: State of the art and clinical perspectives. EXCLI J..

[14] Gondoin A., Grussu D., Stewart D., McDougall G.J. (2010). White and green tea polyphenols inhibit pancreatic lipase *in vitro*. Int. Food Res. J..

[15] Pereira M.N., Justino A.B., Martins M.M., Peixoto L.G., Vilela D.D., Santos P.S., Teixeira T.L., da Silva C.V., Goulart L.R., Pivatto M. (2017). Stephalagine, an alkaloid with pancreatic lipase inhibitory activity isolated from the fruit peel of Annona crassiflora Mart. Ind. Crops Prod..

[16] Belfeki H., Mejri M., Hassouna M. (2016). Antioxidant and anti-lipases activities in vitro of Mentha viridis and Eucalyptus globulus extracts. Ind. Crops Prod..

[17] Birari R., Roy S.K., Singh A., Bhutani K.K. (2009). Pancreatic lipase inhibitory alkaloids of Murraya koenigii leaves. Nat. Prod. Commun..

[18] Pathak N., Rai A.K., Kumari R., Bhat K.V. (2014). Value addition in sesame: A perspective on bioactive components for enhancing utility and profitability. Pharmacogn. Rev..

[19] Tutunchi H., Ostadrahimi A., Saghafi-Asl M. (2020). The effects of diets enriched in monounsaturated oleic acid on the management and prevention of obesity: A systematic review of human intervention studies. Adv. Nutr..

[20] Moreno D.A., Ilic N., Poulev A., Raskin I. (2006). Effects of *Arachis hypogaea* nutshell extract on lipid metabolic enzymes and obesity parameters. Life Sci..

[21] Nor-Liyana J., Siroshini K., Nurul-Syahirah M., Chang W., Nurul-Husna S., Daryl J., Khairul-Kamilah A., Hasnah B. (2019). Phytochemical analysis of *Elateriospermum tapos* and its inhibitory effects on alpha-amylase, alpha-glucosidase and pancreatic lipase. J. Trop. For. Sci..

[22] Khongkarat P., Traiyasut P., Phuwapraisirisan P., Chanchao C. (2022). First report of fatty acids in *Mimosadiplotricha* bee pollen with in vitro lipase inhibitory activity. PeerJ.

